# SIX4 Activation in Inflammatory Response Drives the Transformation of Colorectal Epithelium into Inflammation and Tumor via Feedback-Enhancing Inflammatory Signaling to Induce Tumor Stemness Signaling

**DOI:** 10.7150/ijbs.93411

**Published:** 2024-08-26

**Authors:** Ziyan Zhang, Huang Huang, Lanzhu Peng, Beixian Zhou, Huiling Yang, Zibo Tang, Weiwei Yan, Weifeng Chen, Zhen Liu, Dayong Zheng, Peng Shen, Weiyi Fang

**Affiliations:** 1 Cancer Center, Integrated Hospital of Traditional Chinese Medicine, Southern Medical University, 510315, Guangzhou, China.; 2 Department of Radiotherapy, Sun Yat-sen Memorial Hospital, Sun Yat-sen University, 528406, Guangzhou, China.; 3 Department of gynecology and obstetrics, The Third Affiliated Hospital, Southern Medical University, 510630, Guangzhou, China.; 4 Nursing Department of Nanfang Hospital, Southern Medical University, 510516, Guangzhou, China.; 5 The People's Hospital of Gaozhou, Gaozhou, 525200, China.; 6 School of Pharmacy, Guangdong Medical University, 523808, Dongguan, China.; 7 Department of Radiation Oncology, Shenzhen People's Hospital (The Second Clinical Medical College, Jinan University, The First Affiliated Hospital, Southern University of Science and Technology), Shenzhen, 518020, China.; 8 Guangzhou Municipal and Guangdong Provincial Key Laboratory of Protein Modification and Degradation, School of Basic Medical Sciences, Guangzhou Medical University, Guangzhou, Guangdong, 510095, China.; 9 Shunde Hospital of South Medical University, Foshan City, Guangdong, China.; 10 Kashi first people's Hospital, 844099, Kashi, China.; 11 Department of Oncology, Nanfang Hospital of Southern Medical University, 510515, Guangzhou, China.

## Abstract

Some colorectal cancer patients have experienced normal epithelial transformation into inflammatory and tumor states, but the molecular basis still needs to be further determined. The expression levels of SIX4 are gradually increased in dextran sodium sulfate (DSS) and azoxymethane (AOM)/DSS-induced colonic epithelial inflammation and tumors, respectively, in mice. Targeting SIX4 alleviates intestinal inflammation occurrence and reduces adenoma formation in mice. Clinical sample assays indicated that SIX4 is upregulated in inflammatory bowel disease (IBD) and colorectal cancer (CRC) tissues compared to normal colorectal tissues. In a subsequent study, we found that SIX4, transcriptionally activated by the proinflammatory IL-6/STAT3 signal, binds to c-Jun to transcribe IL-6, thus forming a positive IL-6/STAT3/SIX4/c-Jun feedback loop, which further induces intestinal inflammation occurrence. In addition, elevated SIX4 also induces the expression of DeltaNp63, rather than wild-type p63, by binding to its promoter and thus facilitates the activation of tumor stemness signals, which ultimately leads to the formation of colorectal cancer. Our study first observes that activated SIX4 in inflammation induction drives the transformation of colorectal epithelium into inflammation and tumor, which demonstrates SIX4 as a significant therapeutic target in IBD and colitis-associated colorectal cancer (CAC) and CRC pathogenesis.

## Introduction

Inflammation is a defensive mechanism that facilitates the elimination of pathogens and endogenous substances, thereby eliminating the cause of the disease, eliminating necrotic cells and promoting tissue repair^1^. Nevertheless, in cases where the organism fails to overcome the challenge, there is an increase pro-inflammatory markers in the serum such as IL-1β, IL-6, and TNF-α, leading to the transition of acute inflammation into chronic inflammation^2^. The pathophysiological manifestations of chronic inflammation are intricate and are characterized by tissue remodeling and organ dysfunction^2^. Cancer stem cells (CSCs), which are a subtype of stem-like tumor cells, possess several distinct characteristics such as self-renewal, differentiation potential, drug resistance, and long dormancy^3^. These unique properties enable CSCs to support tumor maintenance and growth, ultimately contributing to tumor recurrence^4^. The presence of an inflammatory environment is recognized as one of the influencing factors fuels tumor stem cell plasticity and alters tumor phenotype and function^4^, ^5^. In addition, numerous studies have found a strong link between inflammation and tumorigenesis^6^, ^7^. The inflammatory environment can facilitate the generation of cells that exhibit characteristics of de novo CSCs, thereby conferring an advantage to CSCs^8^. Inflammatory factors, particularly IL-6, can activate the STAT3/NF-κB pathway in both stromal and tumor cells. This activation plays a crucial role in sustaining the chronic inflammatory state in tumors and inducing the de-differentiation of previously differentiated tumor cells into CSCs^8^, ^9^. Consequently, cells that undergo evolution into CSCs can acquire a competitive edge when confronted with inflammatory pressure.

Colon cancer is the second leading cause of cancer-related mortality worldwide^10^. Numerous studies have provided evidence that supporting the stems of colon cancer cells play an important role in the occurrence, progression, recurrence, metastasis, and resistance to treatment of tumors^11^-^13^. The first evidence of CSCs was found in gastrointestinal tumors and contributed to tumor progression^14^-^16^. Telomerase activity in colon cancer stem cells (CCSCs) situated at the base of the crypt, is more prone to disruption, which can result in an error during the proliferative phase and ultimately lead to tumorigenesis^17^. It should be noted that CCSCs are not exclusively derived from stem cells located in the crypt, but can also arise from colon progenitor cells, tumor cells, or cells that have undergone de-differentiation^18^. These CCSCs are highly proliferative and drug-resistant similar to other CSCs^19^. Chronic inflammation drives the evolution of CCSCs towards more malignant tumor cells^20^. Chronic inflammation is a high-risk factor for tumor development^21^. Patients with chronic IBD, including Crohn's disease (CD) and ulcerative colitis (UC), have a 2-3 times greater risk of developing CAC^22^. Although the incidence of CAC has decreased in recent years, it remains the leading cause of death in patients with IBD^22^-^24^. The pathogenesis of CAC is still not fully understood. Oxidative stress and DNA damage, caused by chronic inflammation, may lead to the over-activation of oncogenes (such as KRAS and MYC) and the inactivation of tumor suppressor genes (such as APC and P53), which are considered as one of the possible reasons^25^, ^26^. Encouragingly, long-term use of NSAIDs significantly reduces the overall risk of CRC patients, indicating that inflammatory cancer transformation is significant in CRC^27^-^29^. However, the role of cancer stem cells in the process of inflammatory cancer transformation remains unclear.

The sine oculis homeobox homolog (SIX) protein family is a group of conserved transcription factors in several different species^30^. The SIX protein family has been reported to promote cancer proliferation, migration and cell stemness in various types of tumors^31^-^33^. SIX4, also known as AREC3, is involved in the process of eye organogenesis, myogenesis and neurogenesis^34^. In cancer studies, He *et al.* found that SIX4 promotes liver cancer metastasis by up-regulating YAP1 and c-Met^35^. Li et al reported SIX4 promotes the progression of osteosarcoma by up-regulating IDH1 and inducing metabolic reprogramming^36^. Sun *et al.* found that SIX4 promotes breast cancer metastasis by activating STAT3^37^; Whereas Aysan *et al.* found that the silence of SIX4 increases sensitivity of melanoma to cisplatin^38^. Although there have been studies based on public databases that predict a potential carcinogenic role of SIX4 in CRC^39^. However, the role and mechanism of SIX4 in IBD as well as the transformation of inflammation to cancer have not been reported.

In this study, we developed a mouse model of colitis-associated colon cancer(CAC) using AOM/DSS treatment. Through high-throughput transcriptome sequencing, we observed a significant upregulation of SIX4 in the colon tissue of mice following AOM/DSS treatment. Further, we observed that targeting SIX4 attenuated the transformation of inflammation to cancer in intestinal epithelium via inactivating IL-6/STAT3/SIX4/c-JUN feedback loop, which subsequently suppressed DeltaNp63-mediated tumor stemness signals. Our data demonstrated that SIX4 is a key therapeutic target in CAC.

## Materials and methods

### Animal models and the assessment of disease activity index (DAI)

The Ethics Committee of Southern Medical University Affiliated Integrated Hospital of Traditional Chinese Medicine approved all animal experiments (2021-0096). All mice used in this study were five to six weeks old BALB/c mice and were housed in a specific pathogen-free (SPF) environment. The IBD/CAC model was divided into three groups: Negative control (NC), DSS and AOM +DSS. NC: No special treatment; DSS: on the fifth day, DSS was administered in their drinking water for a period of 5 days, with 14-day intervals and 3 cycles; AOM+DSS: single intraperitoneal injection of AOM (10 mg/kg) followed by the same treatment as the DSS group^40^. *In vivo* siRNA therapy was initiated on day 43 of the animal experiment by intraperitoneal injection with 30 μg of control siRNA, siSIX4 (RQCON Biological Technology Co., Ltd. Xi'an, China) formulated with *in vivo*-jetPEI (Polyplus Transfection, Illkirch, France) three times a week^41^. Body weight loss, stool consistency, and occult blood were monitored daily. The disease activity index (DAI) was calculated as previously described^42^. In summary, the calculation involved the utilization of the following parameters: body weight loss (0, none; 1, <5%; 2, 5%-10%; 3, 10%-20%, 4, >20%), diarrhea (0 points for normal stools, 2 points for loose stools, and 4 points for watery diarrhea) and hematochezia (0 points for no bleeding, 2 points for slight bleeding, and 4 points for gross bleeding). The DAI score represents the cumulative value of three indicators, ranging from 0 to 12 points. After the experiment, the mice were subjected to euthanasia. The colons were collected, measured, photographed, and stored appropriately for future experiments.

### Immunohistochemical staining and hematoxylin and eosin (HE) staining

Immunohistochemical staining was according to standard protocols. In brief, the tissue sections underwent dewaxing followed by boiling in EDTA buffer for antigenic repair. After inhibiting peroxidase, the section was subsequently blocked in 5% goat serum at room temperature (RT) for 30 min. The primary antibody was incubated at 4℃ overnight, followed by sequential incubation of the secondary antibody for 1h at RT sequentially. Finally, staining was performed using DAB and hematoxylin (Solarbio, Beijing, China). Scoring was done according to a previously described method^43^. The immunohistochemical scores ranged from 0 to 12. In the tissue microarray analysis, the median score of the samples was 5. Based on this score, the samples were categorized into two groups: high-expression (scores ≥ 5) and low-expression (scores < 5) groups. Hematoxylin and eosin (HE) staining was conducted using established protocols. In short, slides were dewaxed using xylene and gradient alcohol solutions, followed by treatment with hematoxylin for 5 min, hydrochloric acid alcohol differentiation was performed for 1s, flushing with water for 5 min, and eosin was applied for 1 min. After dehydration, the slices were subsequently sealed.

### Microarray

In summary, the total RNA of the sample was collected for quantification and subsequently examined to verify its integrity. Then, total RNA was transcribed to double strand cDNA, then synthesized into cRNA and labeled with Cyanine-3-CTP. The labeled cRNAs were subjected to hybridization on the microarray. After washing process, the arrays were scanned by the Agilent Scanner G2505C (Agilent Technologies). Finally, raw data were obtained using the Feature Extraction software, and these raw data were normalized using the quantile algorithm in the Genespring software.

### Reverse transcription-real-time quantitative PCR (RT-qPCR)

Total RNA from cells and animal tissue was extracted using the Total RNA Isolation Kit (Foregene, Chengdu, China). mRNA was transcribed into cDNA by Reverse Transcription Reagent Kit (Vazyme, Nanjing, China). The resulting cDNA served as a template to amplify target genes in subsequent qPCR tests. Quantitative real-time PCR was performed using the CFX96 Real-Time System (Bio-Rad). Primers are shown in the supplementary materials (Supplementary Table 1).

### Western blotting and antibodies

Western blotting was performed according to standard protocols. Antibodies of SIX4 (21305-1-AP), CD133 (66666-1-Ig), IL-6 (21865-1-AP), JAK1 (66466-1-Ig), and p63 (60332-1-Ig) were purchased from Proteintech (Rosemont, IL, USA). Anti-CD44 (3570S) and Anti-C-JUN (9165S) were purchased from Cell Signaling Technology (Danvers, MA, USA). Anti-flag(F1804) was purchased from Sigma (St. Louis, MO, USA); Anti-GAPDH (AP0063) was purchased from Bioworld (Bloomington, MN, USA). Anti-STAT3 and Anti-p-STAT3 were purchased from Immnoway (Plano, TX, USA). Anti-SOX2 (ab97959) was purchased from Abcam (Cambridge, MA, USA). Western blotting was performed by Mini-PROTEAN Tetra- and Mini Trans-Blot (Bio-Rad, Hercules, CA, USA). The images were captured with Chemiluminescence Imaging System (Minichemi, Beijing, China).

### Cell culture and reagents

NCM460, SW480, SW620, HT29, and HCT116 were cultured in RPMI 1640 Medium (Vivacell, Shanghai, China) containing 10% fetal bovine serum (FBS) (Biowest, Nuaillé, France) at 37°C under 5% CO_2_. Culture dishes and centrifuge tubes were purchased from Jet Biofil (Guangzhou, China). Recombinant human IL-6 was purchased from PeproTech (Cranbury, NJ, USA).

### Clinical samples tissue

Tissue microarray (TFcolon-01) was purchased from TUFEI Biotech Company (Shanghai, China). The use of clinical materials was approved by the Ethics Committees of TUFEI Biotech Company (20210156).

### Transfection and lentivirus infection

SIX4 siNC/siRNA and plasmid DNA containing SIX4 were transfected into colon cancer cells using the Lipofectamine 3000 Transfection Reagent (Invitrogen, Carlsbad, CA, USA) according to the manufacturer's instructions. Stable cell lines expressing exogenous shNC/shSIX4 in SW480 and HT29 were constructed using lentiviruses (Genechem, Shanghai, China) labeled with green fluorescent protein (GFP). The multiplicity of infection (MOI) was set to 20. Three days post-infection, GFP fluorescence was detected, and the cells were screened using 8 ng/ml of puromycin. The expression level of SIX4 in SW480 and HT29 cell lines stably infected with lentiviruses was assessed by Western blot analysis.

### Tumorspheres formation assay

Cells were (2000 cells / well) seeded in 6-well ultra-low-attachment culture plates. The cells were propagated in serum-free DMEM/F12 (Gibico, Waltham, Mass, USA) with 20 ng/ml epidermal growth factor (EGF, PeproTech), 20 ng/ml fibroblast growth factors (FGF, PeproTech), and 2% B27 (ThermoScientific). The medium was changed every 4 days. After 2-3 weeks of incubation, tumorspheres were observed and photographed.

### Colony formation assay

Cells were seeded in 6-well culture plates at 200 cells/well and incubated for two weeks at 37 °C in 5% CO_2_ atmosphere. The medium was changed every 4 days. After incubation, colonies were washed twice with phosphate buffer saline (PBS), fixed with 4% formaldehyde, and stained with Giemsa.

### Flow cytometry

A total of 10^6^ cells were harvested 48 hours after transfection and then washed twice with PBS. The cells were resuspended in binding buffer (PBS containing 1% FBS). Subsequently, anti-CD44-FITC (338803, Biolegend, San Diego, CA, USA) and anti-CD133-APC (397905, Biolegend) were added and the cells were incubated for 15-20 minutes at room temperature (RT), protected from light. After washing the cells twice with binding buffer, a FACS caliber flow cytometer (BD Biosciences, NJ, USA) was used to analyze them.

### Tumor xenograft experiments

Four- to five-week-old nude mice were purchased from SPF (Beijing) Biotechnology Co., Ltd. (Beijing, China). Cells (SW480-shNC, SW480-shSIX4) with varying quantities (5 × 10^6^, 2 × 10^6^, 1 × 10^6^, 5 × 10^5^) were suspended in 100 µl of PBS (containing a 20% concentration of matrigel, BD Biosciences), respectively. After 28 days, the mice were euthanized, and the tumors were weighed, photographed, dehydrated, and finally embedded in paraffin.

### Immunofluorescence staining (IF)

Cells were harvested 48 h after transfection, seeded into confocal imaging dishes for 12 h, and subsequently rinsed twice with PBS. The cells were then fixed with 4% paraformaldehyde and 2 mg/mL glycine for 15 min and permeabilized with 0.2% Triton X-100 for 5 min. After blocking with 5% FBS for 30 min and incubating with specific primary antibodies at 4°C overnight, the cells were rinsed three times with PBS and stained with Alexa Fluor secondary antibodies (Earthox, Millbrae, CA, USA) and DAPI (Beyotime, Biotechnology).

### Co-immunoprecipitation assay (Co-IP)

Treated cells were subjected to immunoprecipitation lysis buffer (Thermo Scientific, Waltham, Mass, USA). Cell lysates were exposed to specific antibodies or Normal IgG (final concentration of 20 μg/mL) at 4 °C overnight in the rotator. Cell lysates containing antibody was added to the pre-washed protein A/G magnetic beads (Bimake, Shanghai, China) and incubated for 30 min at RT. The pellet underwent five washes using a washing buffer (50 mM Tris, 150 mM NaCl, 0.2% TritonX-100, 0.2%Tween, pH 7.5) five times. The bound proteins were resolved in 5× SDS loading buffer and boiled for 10 min at 95 ℃ and subjected to immunoblotting.

### Chromatin immunoprecipitation (ChIP) assay

The experiments were performed using the Pierce Magnetic ChIP Kit in accordance with the manufacturer's manual (Thermo Scientific, Waltham, MA, USA). Briefly, the cells were cross-linked using a final concentration of 1% formaldehyde. The cross-linked cells were treated with a membrane extraction buffer and MNase to remove membrane proteins and expose the nucleic acid. 2 μg of each of C-JUN, STAT3, SIX4, and normal rabbit IgG antibody were added to the supernatant containing the digested chromatin, respectively. For both the experimental group and the control group, IP reactions were incubated overnight at 4 °C with mixing. After incubation, ChIP-grade protein A/G magnetic beads were introduced into each IP reaction and further incubated for 2 h at 4 °C with mixing. The beads were then cleaned, and the nucleic acid-protein complex was eluted from the beads. Proteinase K was used to remove the proteins from the nucleic acid-protein complex. Finally, DNA fragments were purified after being washed in a DNA clean-up column.

### Dual-Luciferase reporter assay

Based on the SIX4/p63 promoter sequence predicted by JASPAR, either the wild-type (WT) or mutant SIX4/p63 promoter sequence was cloned into the psicheck-2 vector. Co-transfect the WT or mutant vector, along with STAT3/SIX4 overexpression and control vectors, into cells, and measure the transfection activity 48 hours after luciferase activation using the Dual-Luciferase reporter assay system (Promega, Madison, WI, USA). Mutation details are shown in Figure 5F and Figure 7B.

### Statistical analysis

Statistical analyses were performed using IBM SPSS Statistics 25 (IBM, Armonk, NY, USA). Paired samples or two independent samples of grade data were tested using nonparametric tests. For continuous variables conforming to normal distribution, two independent samples were analyzed using the t-test, and multiple groups were analyzed using one-way ANOVA. Survival plots were generated using the log-rank test and the Gehan-Breslow-Wilcoxon test. Cox regression was employed to screen for single and multiple risk/protective factors. All statistical tests were two-sided, and the asterisk indicated statistical significance. *P < 0.05, **P < 0.01, ***P < 0.001.

### Availability of data and materials

The STAT3 ChIP data from the ENCODE (ENCSR000DOQ, Kevin Struhl, HMS laboratory, Michael Snyder, Stanford), and we downloaded the call sets from the ENCODE portal (https://www.encodeproject.org/) with the following identifiers: ENCFF009TFU, ENCFF994RVN, ENCFF282DRU, ENCFF327LFX, ENCFF969CSL, ENCFF987HQY. For detailed sequencing data, please refer to the Supplementary Materials.

## Results

### SIX4 is increased in colitis-induced colorectal cancer animal model

**Figure 1:**
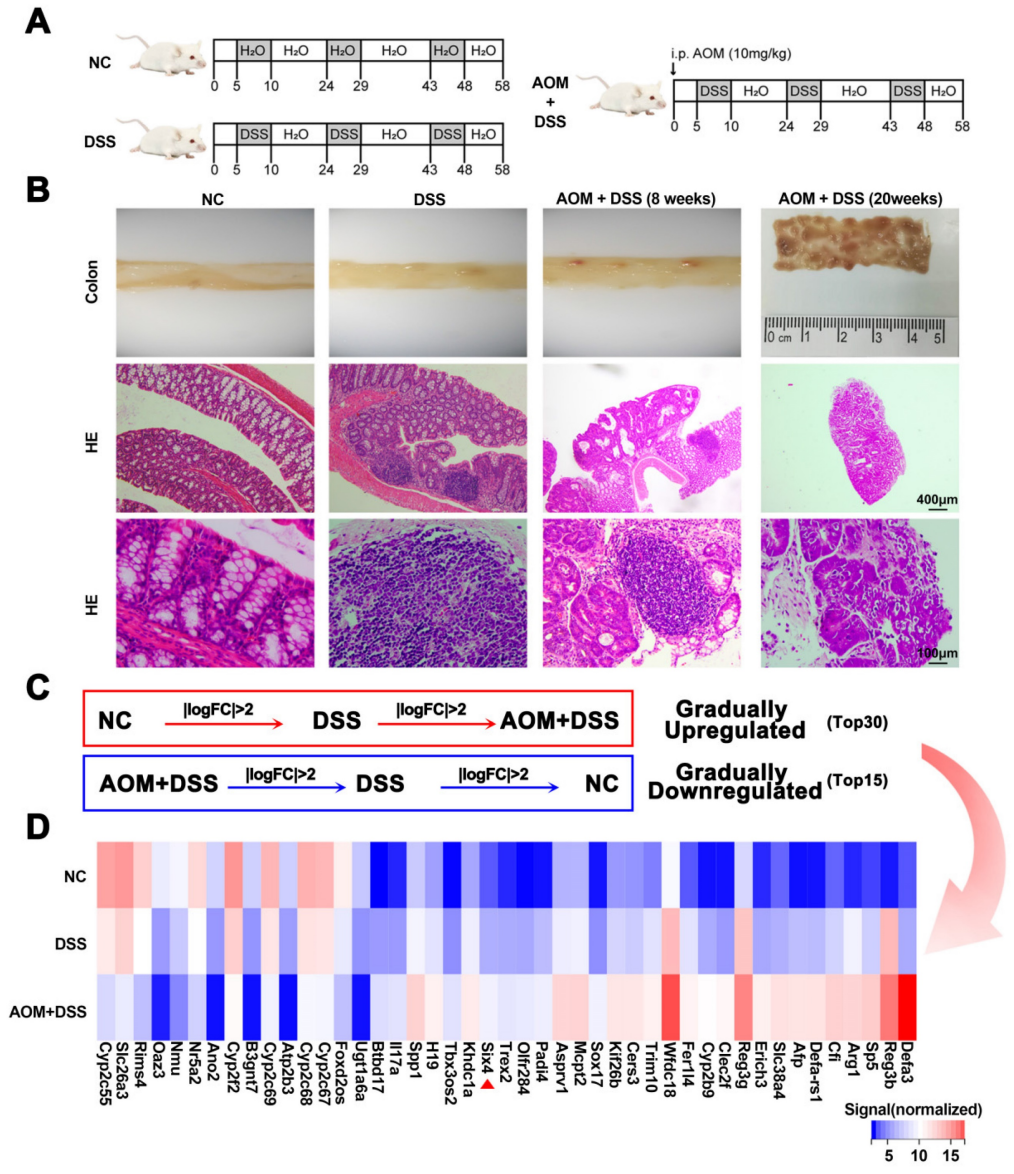
** SIX4 is an important factor in the normal-inflammation-cancer transformation.** (A) Construction of mouse models of IBD and CAC. NC: normal drinking water; DSS: Starting from day 5, 1.5% DSS was added to drinking water for 7 consecutive days, followed by normal drinking water for 14 days, and the DSS-water cycle was repeated for 3 times. AOM+DSS: Mice were first intraperitoneally injected with 12mg/kg AOM, and subsequent treatment was consistent with DSS group. (B) Representative images of colon tissue and its HE staining. (C) Differentially expressed genes that were gradually upregulated or downregulated were screened from NC group to DSS group to AOM+DSS group. *P*<0.05, |LogFC|>2. (D) Heat map of differentially expressed genes in (C).

This study established DSS and AOM/DSS mouse models to explore which genes play a key role in colitis-associated colon cancer (Figure 1A). Subsequently, the mice were euthanized. Pathological analysis confirmed that these models were successfully constructed (Figure 1B). We harvested intestinal tissue for transcriptomic sequencing. Through a stepwise screening process, genes that were gradually upregulated and downregulated were filtered (Figure 1C-D). Based on the TCGA database, we presented the expression of these differential genes between colorectal cancer and normal tissues, as well as patient survival data. Among the identified genes, SP5, SIX4, TREX2, and SPP1 were upregulated and were adverse prognostic factors in colorectal cancer. Conversely, B3GNT7 and GAL3ST2 were downregulated and were protective factors in colorectal cancer (Supplementary Figure 1-2).

### Inhibition of SIX4 alleviates intestinal inflammation and reduces adenoma formation

**Figure 2:**
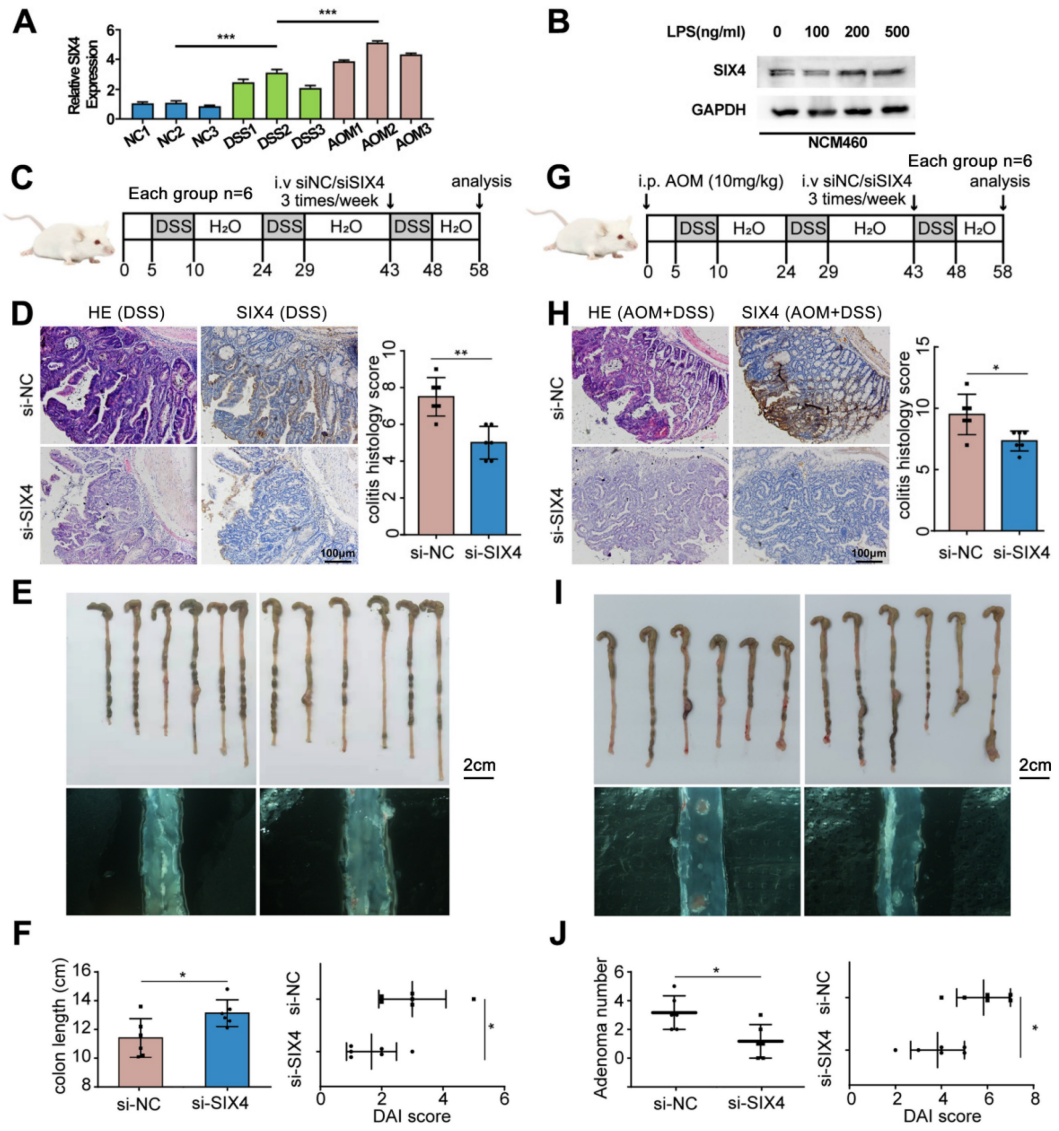
** Inhibition of SIX4 alleviated intestinal inflammation and reduced adenoma formation.** (A) mRNA expression level of SIX4 in colon tissues of mice treated with different treatments. AOM: AOM+DSS. (B) Expression of SIX4 in human normal colonic epithelial cell line NCM460 treated with LPS at different concentrations. GAPDH served as a loading control (C) Starting from day 5, 1.5% DSS was added to drinking water for 7 consecutive days, followed by normal drinking water for 14 days, and the DSS-water cycle was repeated for 3 times. Starting from day 43, siNC / siSIX4 was injected intraperitoneally 3 times a week for a total of 6 injections. (D) Representative images of colonic mucosa tissue HE staining and SIX4 IHC staining as well as the IHC staining score of SIX4. (E) Representative images of colon tissues. (F) Statistical analysis of colon length in colitis. And the DAI score of DSS + siNC / siSIX4 treated mice. (G) Mice were first intraperitoneally injected with 12mg/kg AOM, and subsequent, 1.5% DSS was added to drinking water for 7 consecutive days, followed by normal drinking water for 14 days, and the DSS-water cycle was repeated for 3 times. Starting from day 43, siNC/siSIX4 was injected intraperitoneally 3 times a week for a total of 6 injections. (H) Representative images of colonic mucosa tissue HE staining and SIX4 IHC staining as well as the IHC staining score of SIX4. (I) Representative images of colon tissues. (J) Statistical analysis of adenomas number in CAC. And the DAI score of DSS +AOM +siNC/siSIX4 treated mice.

In order to further verify the screened differential genes, the mRNA expression of SP5, SIX4, TREX2, SPP1, B3GNT7, and GAL3ST2 in the intestinal tissues of different groups of mice was examined (Supplementary Figure 3). The results showed that the mRNA expression levels of TREX2, SIX4, SPP1, and GAL3ST2 were consistent with the sequencing results. The selection of SIX4 was based on its pronounced differential expression (Figure 1D, Figure 2A), as well as its strong correlation with patient survival (Supplementary Figure 1). Furthermore, we analyzed the expression of SIX4 in NCM460, a normal intestinal epithelial cell line, following lipopolysaccharide (LPS) treatment. The results showed that SIX4 was indeed upregulated during inflammation (Figure 2B). To investigate the role of SIX4 in the development of CAC in a mouse model induced by AOM/DSS, *in vivo*-optimized si-NC and si-SIX4 were used to treat the CAC mouse model (Figure 2C, 2G). The efficiency of the siRNA sequences has been assessed using murine-derived cells (Supplementary Figure 4A). Additionally, the efficacy of siSIX4 treatment was validated in mice (Figure 2D, 2H, Supplementary Figure 4B). Based on histomorphological analysis, we found that the use of si-SIX4 increased colon length in DSS-treated mice and reduced the number of adenomas induced by AOM+DSS. Moreover, the DAI score, based on the assessment of stool consistency, bloody stool, and weight loss, indicated that the use of siSIX4 alleviated DSS/AOM-induced colitis/adenoma (Figure 2E, 2F, 2I, 2J).

### SIX4 is upregulated in IBD as well as colorectal cancer and is a poor prognostic factor

**Figure 3:**
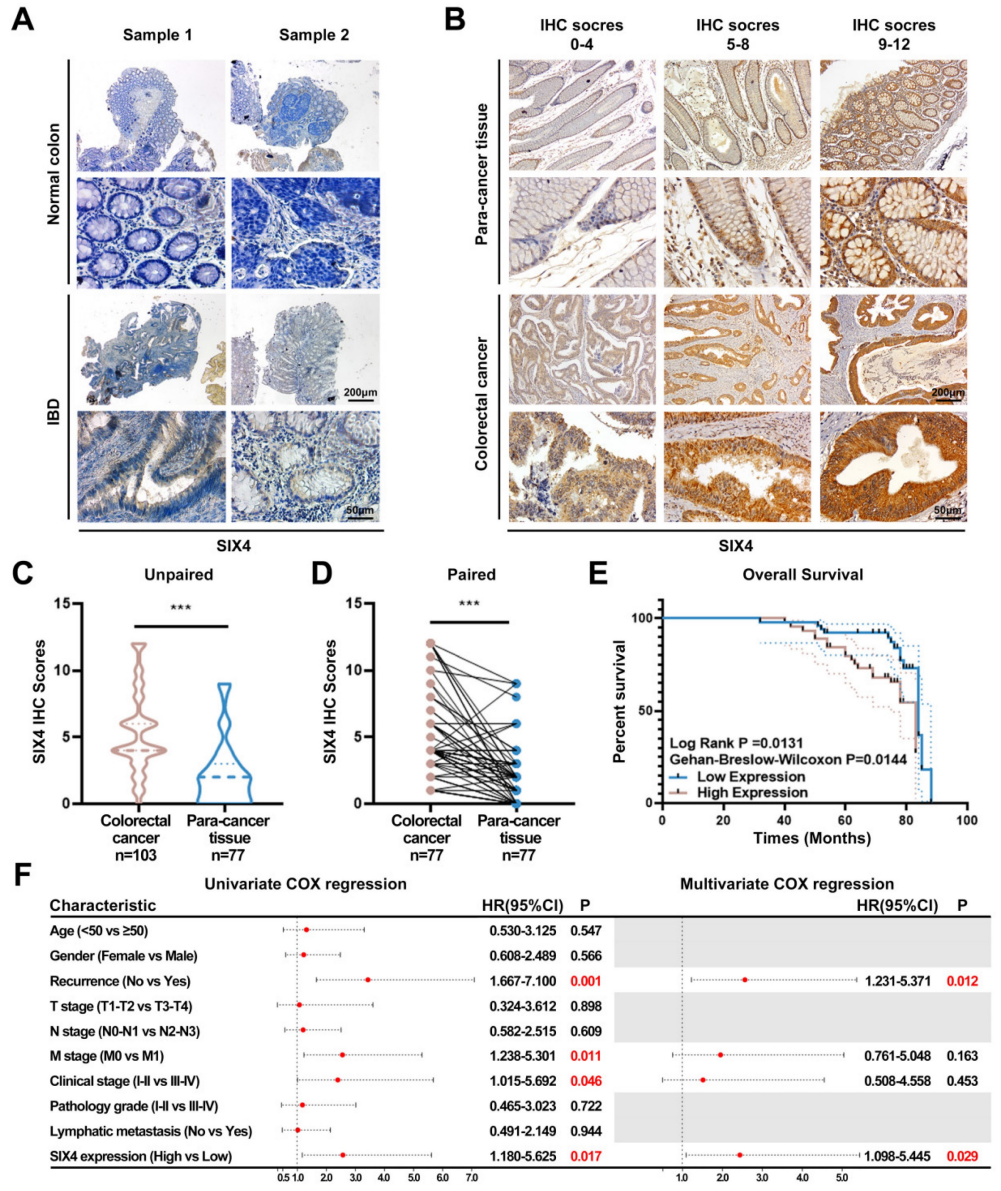
** SIX4 was upregulated in IBD and colorectal cancer and is a poor prognostic factor.** (A) Expression of SIX4 in normal and IBD colon tissues. Scale bars, 200 μm or 50 μm (B) Expression of SIX4 in para-cancer tissues and colorectal cancer tissues as well as its. Scale bars, 200 μm or 50 μm (C) unpaired and (D) paired statistical analysis. (E) Overall survival in patients with different SIX4 expressions. The median score of 4 was taken as the cut off. SIX4 high expression: IHC score>4; SIX4 low expression: IHC score ≤4. (F) Summary of univariate and multivariate Cox regression analysis of overall survival duration. The data of (B-F) are generated from tissue microarrays.

To elucidate the potential value of SIX4 in IBD and CAC, immunohistochemistry was employed to assess the expression of SIX4. The expression of SIX4 was upregulated in IBD tissues compared to the normal colon tissues (Figure 3A). In tissuearray (Figure 3B), SIX4 was significantly upregulated in colorectal cancer when compared to the para-cancer tissue (Figure 3C-D). Based on the detailed clinical data associated with the IHC scores, we analyzed the relationship between SIX4 expression and clinical pathological characteristics using one-way ANOVA analysis (Table 1). The overall survival showed that patients with low SIX4 expression have longer survival time (Figure 3E). In addition, the Univariate Cox regression analysis showed that tumor recurrence (*P* = 0.001 HR = 3.44 95%CI = 1.667-7.100), M stage (*P* = 0.011 HR = 2.562 95%CI = 1.238-5.301), clinical stage (*P* = 0.046 HR = 2.403 95%CI = 1.015-5.692), and the expression of SIX4 (*P* = 0.017 HR = 2.577 95%CI = 1.180-5.625) were associated with the patient survival. Multivariate Cox regression analysis showed that tumor recurrence (*P* = 0.012 HR = 2.571 95%CI = 1.231-5.371) and the expression of SIX4 (*P* = 0.029 HR = 2.445 95%CI = 1.098-5.445) were identified as the independent risk factors (Figure 3F).

### Inhibition of SIX4 suppresses colorectal cancer stemness

**Figure 4:**
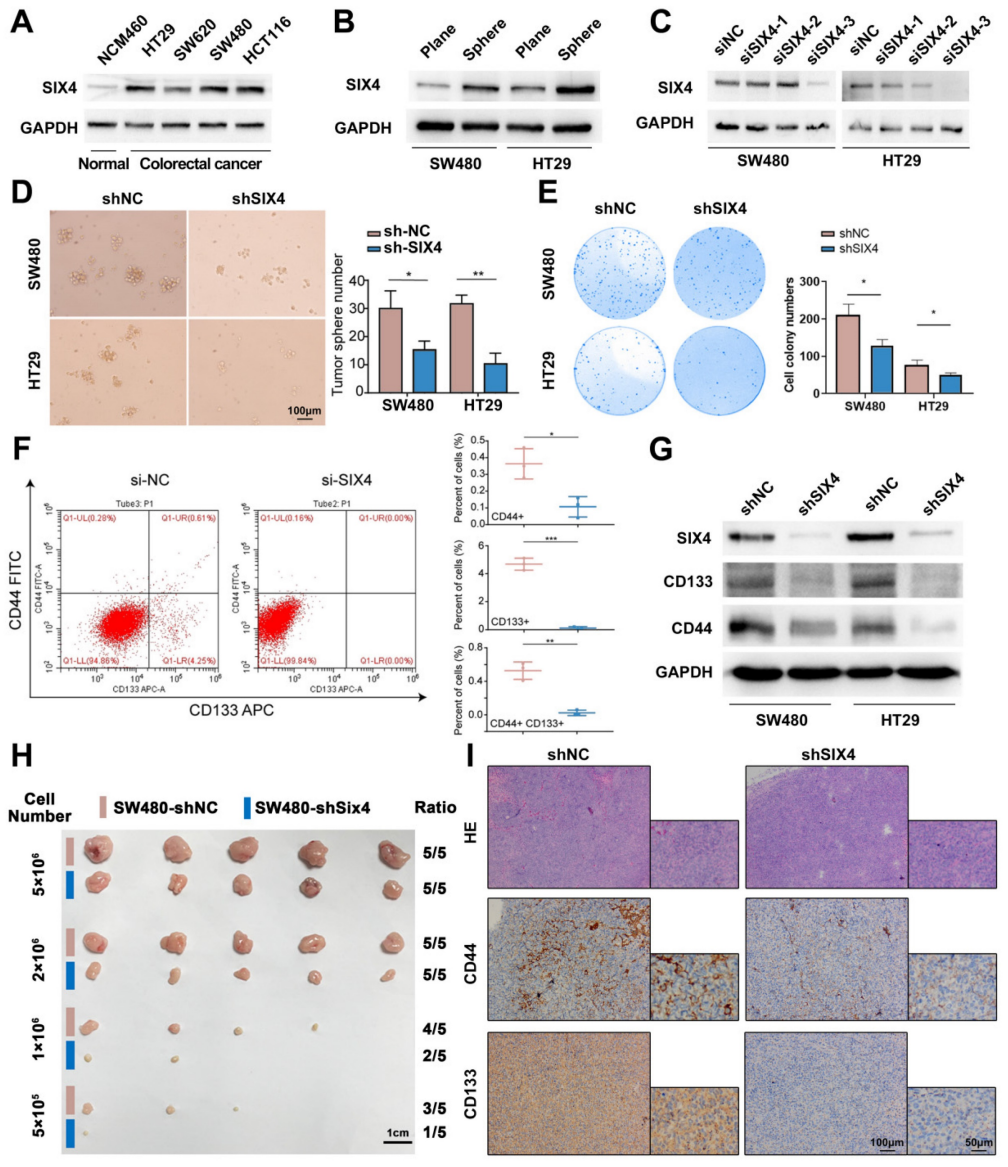
** Inhibition of SIX4 suppresses colorectal cancer stemness.** (A) Expression of SIX4 in normal and colorectal cancer cells. (B) Expression of SIX4 of tumor spheres models and adherent cells in SW480 and HT29 cells. (C) The knockdown efficiency of SIX4 in SW480 and HT29 cells. (D) and (E) shNC or shSIX4 cell lines were used to perform tumorspheres assay and clonal formation assay. (F) Flow cytometry analyses of si-SIX4 cells and control cells. (G) Western blotting showing changes in proteins of stemness markers in SW480 and HT29 with or without SIX4. (H) and (I) Nude mice xenograft tumor model constructed by injecting gradient concentrations of tumor cells (SW480-shNC and SW480-shSIX4) into two sides of the mice's backs. Xenografts were stained with H&E and subjected to immunohistochemistry for CD44 and CD133 expression. Scale bars indicate 1cm for tumor nodules. Scale bars indicate 100μm for H&E staining and immunohistochemistry.

Firstly, we compared the expression of SIX4 in four colorectal cancer cell lines as well as one normal intestinal epithelial cell line and found that SIX4 was upregulated in colorectal cancer cell lines (Figure 4A). Furthermore, we constructed tumor spheres models of two colorectal cancer cells and compared them with adherent cells. The result showed that SIX4 was significantly upregulated in tumorsphere formation (Figure 4B). Therefore, we speculated that SIX4 may be related to tumor stemness. To verify the functional significance of SIX4 in colorectal cancer, three siSIX4 sequences were used to knockdown SIX4. SiSIX4-3 fragment has the highest knockdown efficiency and subsequently was selected to establish a lentivirus-mediated SIX4 stable knockdown cell line (Figure 4C). Tumorspheres assay and clonal formation assay showed that the knockdown of SIX4 decreased tumor spheres and clone formation (Figure 4D-E). To mitigate the potential interference of GFP fluorescence in the stable knocked down SIX4 cells, si-SIX4 was used to transiently transfect colorectal cell. Flow cytometry analysis showed that the knockdown of SIX4 inhibited the expression of CD133 and CD44 (Figure 4F). Consistent with this, western blot analysis showed that the knockdown of SIX4 downregulated the expression of CD133, CD44, and p63 (Figure 4G).

In the nude mice xenograft tumor model, a concentration gradient of tumor cells (SW480-shNC and SW480-shSIX4) was injected subcutaneously into two sides of the mice's backs. By observing the tumor-forming ratio, we found that SIX4 knockdown cell lines required higher population density to form tumor nodules (Figure 4H). In addition, immunohistochemistry of tumor nodules showed that the knockdown of SIX4 decreased the expression of CD44 and CD133 (Figure 4I).

### SIX4 is activated by the IL-6/STAT3 signal

**Figure 5:**
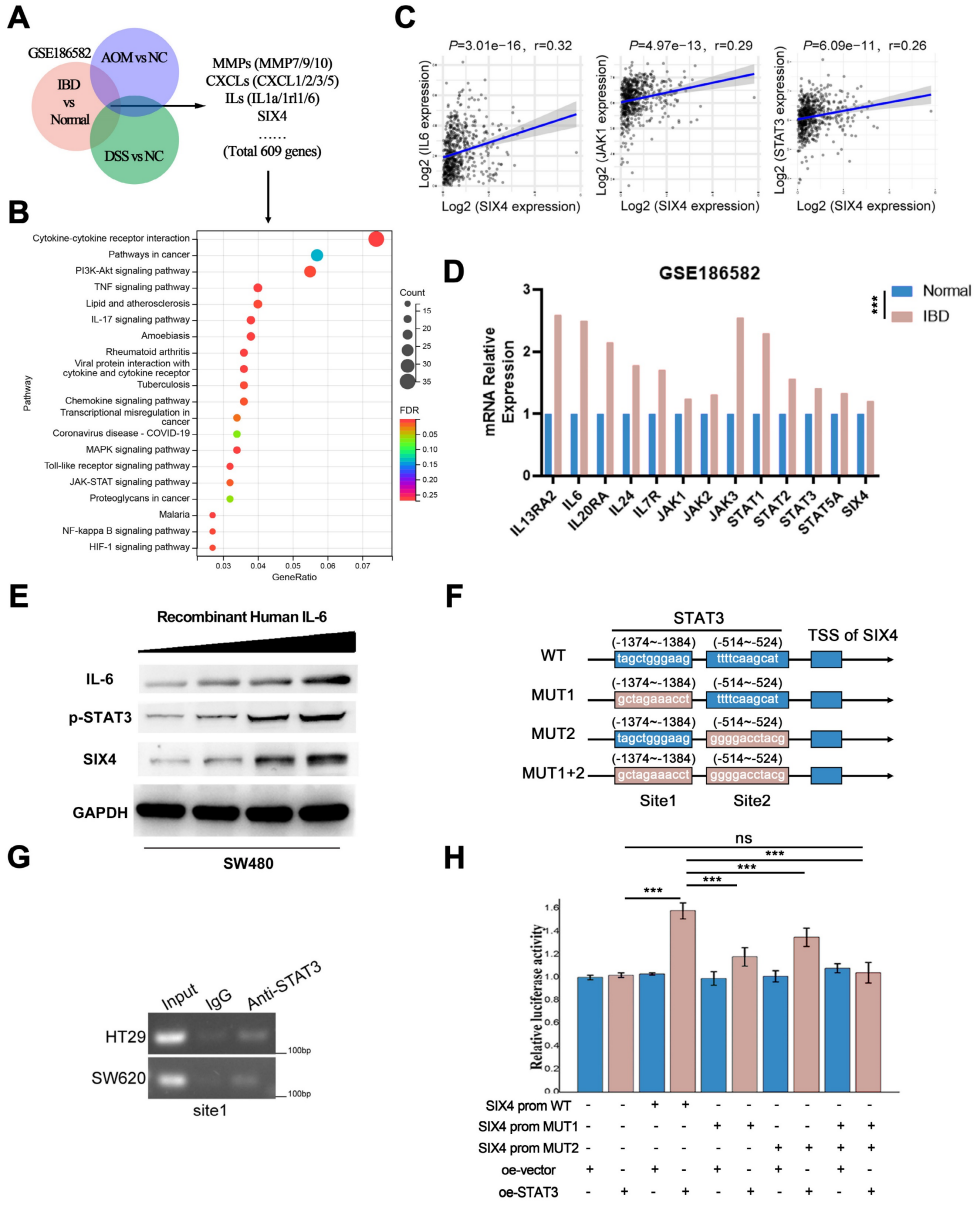
** SIX4 is activated by the IL-6/STAT3 signal.** (A) After intersection,609 differential genes were identified based on the Venn diagram. (B) KEGG analysis: horizontal coordinate is the gene ratio; vertical coordinate shows the pathway terms; node size indicates the number of genes enriched in the pathway; node color indicates FDR, *P<*0.05. (C) Studies from TCGA database showed the correlation between SIX4 mRNA expression and IL-6, JAK1, STAT3's mRNA. (D) Multiple target genes of the IL-6/STAT3 signaling pathway were shown in bar chat. (E) Western blotting showing changes in proteins of IL-6, p-STAT3, and SIX4 after stimulating by IL-6. (F) Diagram of wild-type and mutant of STAT3 combined with SIX4 promoter region. (G) Chromatin immunoprecipitation results were validated by agarose gel electrophoresis analysis. (H) Luciferase reporter assays were conducted to assess STAT3 binding to the promoter of SIX4 with or without mutant.

To further explore the mechanism of SIX4 involved in inflammation and tumor stemness, we compared the mice sequencing results (DSS vs NC and AOM+DSS vs NC) with the clinical sequencing results (IBD vs Normal, GSE186582) and screened out 609 differential genes (Figure 5A). Given the inherent limitations of the mouse model in fully replicating human disease, we opted to identify more significant differentially expressed genes by comparing the sequencing data from mice with that of clinical samples. The enrichment analysis of the KEGG pathway suggested that the cytokine-cytokine receptor interaction, PI3K-AKT signaling pathway, TNF signaling pathway, IL-17 signaling pathway, and so on were changed in IBD (Figure 5B). Interestingly, despite not being the most highly ranked pathway in terms of enrichment, the IL-6/STAT signaling pathway plays a significant role in various pathways such as cytokine-cytokine receptor interaction, TNF signaling pathway, IL-17 signaling pathway, and MAPK signaling pathway. These pathways have been found to activate IL6 transcription (Supplementary Figure 5). Therefore, we suspected that SIX4 is activated via IL-6/STAT3 signal. The analysis of the TCGA database showed that the expressions of IL-6 and STAT3 were positively correlated with SIX4 expression (Figure 5C). Multiple target genes of the IL-6/STAT3 signaling pathway were upregulated in the clinical samples (Figure 5D). We have also observed that the expression of SIX4 was stimulated by recombinant human IL-6 (Figure 5E). Analysis of the transcription factor ChIP-seq Clusters from ENCODE database showed that STAT3 has a binding signal in the SIX4 promoter region in normal mammary epithelial cells (Supplementary Figure 6A). In addition, STAT3 was predicted as the transcription factor of SIX4. JASPAR database analysis reveals the presence of potential binding regions for STAT3 in the promoter region of SIX4 (Figure 5F WT, Supplementary Figure 6B). The results of chromatin immunoprecipitation were analyzed using agarose gel electrophoresis, which revealed the binding of STAT3 to the promoter region of SIX4 (Figure 5G). Further, we mutated the promoter region of SIX4 (Figure 5F, MUT). The dual-luciferase reporter system revealed that the overexpression of STAT3 induces the expression of SIX4. Mutating a single site partially inhibited the expression of SIX4, whereas mutating two sites completely abolished the expression of SIX4 induced by overexpression of STAT3 (Figure 5H).

### SIX4 recruits C-JUN prompting STAT3 nuclear transposition to activate the IL-6/ STAT3 signal and induce self-expression through a positive feedback loop

**Figure 6:**
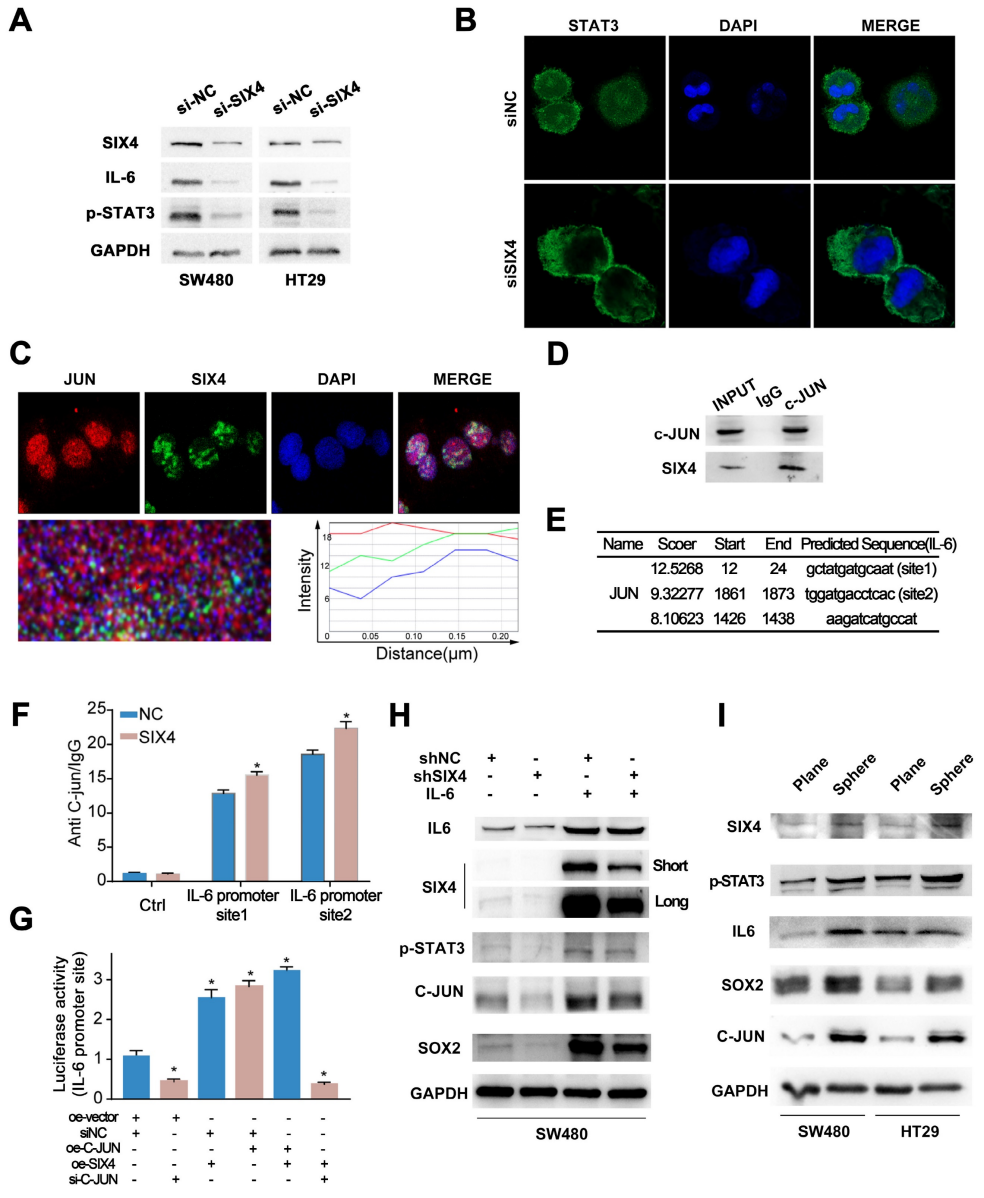
** SIX4 recruits C-JUN prompting STAT3 nuclear transposition to activate the IL-6/STAT3 signal and induce self-expression through a positive feedback loop.** (A) The protein expression of SIX4, IL-6, and p-STAT3 in in si-NC and si-SIX4 colon cancer cells were examined by western blot. (B) Immunofluorescence of STAT3 in si-NC and si-SIX4 cells (C) Immunofluorescence of JUN and SIX4 in colon cancer cells. (D) Co-IP assay of C-JUN and SIX4. The input was diluted as 1/10. (E) Three C-JUN binding sites in the promoter of IL-6 were predicted via JASPAR. (F) ChIP assays were performed to asses C-JUN binding to the promoter of IL-6 in NC and SIX4-overexpression cells. (G) The luciferase reporter gene assay was used to evaluate the effect of SIX4 overexpression on C-JUN-mediated IL-6 expression. (H) Western blot experiments were used to detect the expression of IL-6, SIX4, p-STAT3, C-JUN, SOX2 in SW480 cells with NC or SIX4 knockdown adding recombinant human IL-6 respectively. (I) Western blot experiments were used to detect the expression of SIX4, p-STAT3, IL-6, SOX2, and C-JUN in tumor spheres compared to adherent cells.

Although we preliminarily demonstrated that SIX4 is activated by the IL-6/STAT3 signaling pathway, the mechanism remains unclear. We found that the knockdown of SIX4 downregulated the IL-6, STAT3 and inhibited the phosphorylation of STAT3 (Figure 6A). Further, we performed immunofluorescence experiments and found that the knockdown of SIX4 inhibited the nuclear transposition of STAT3 (Figure 6B). Strikingly, based on the BioGrid database, C-JUN (also named JUN, AP-1) was the potential interacting protein with SIX4. First, the immunofluorescence co-focused experiment showed SIX4 and C-JUN were confocal in cell nuclear (Figure 6C). Subsequently, the co-immunoprecipitation experiment was performed to verify the interaction between SIX4 and C-JUN, and the result confirmed their interaction (Figure 6D). In addition, C-JUN as a transcription factor regulates IL-6 expression. Therefore, we speculated that the binding of SIX4 with C-JUN facilitates the transcription of IL-6. To confirm this, we used JASPAR to predict the binding sequence of C-JUN in the IL-6 promoter region (Figure 6E), and performed the chromatin immunoprecipitation. The result showed that the overexpression of SIX4 increased the binding of C-JUN to the IL-6 promoter (Figure 6F). In addition, the dual-luciferase reporter system revealed that the overexpression of SIX4 or C-JUN both promoted the expression of IL-6, but the knockdown of C-JUN in SIX4 overexpressing cells blocked this promotion (Figure 6G). This suggested that there may be positive feedback loops in the SIX4 and IL-6/STAT3 signaling pathways. Rescue experiments found that the introduction of recombinant human IL-6 reversed the inhibition of STAT3, C-JUN, SOX2 by SIX4 knockdown (Figure 6H). In addition, SIX4, STAT3, IL-6, SOX2, and C-JUN were upregulated in tumor spheres compared to adherent cells (Figure 6I).

### SIX4 promotes the stemness of colon cancer cells via regulating p63 transcription

**Figure 7:**
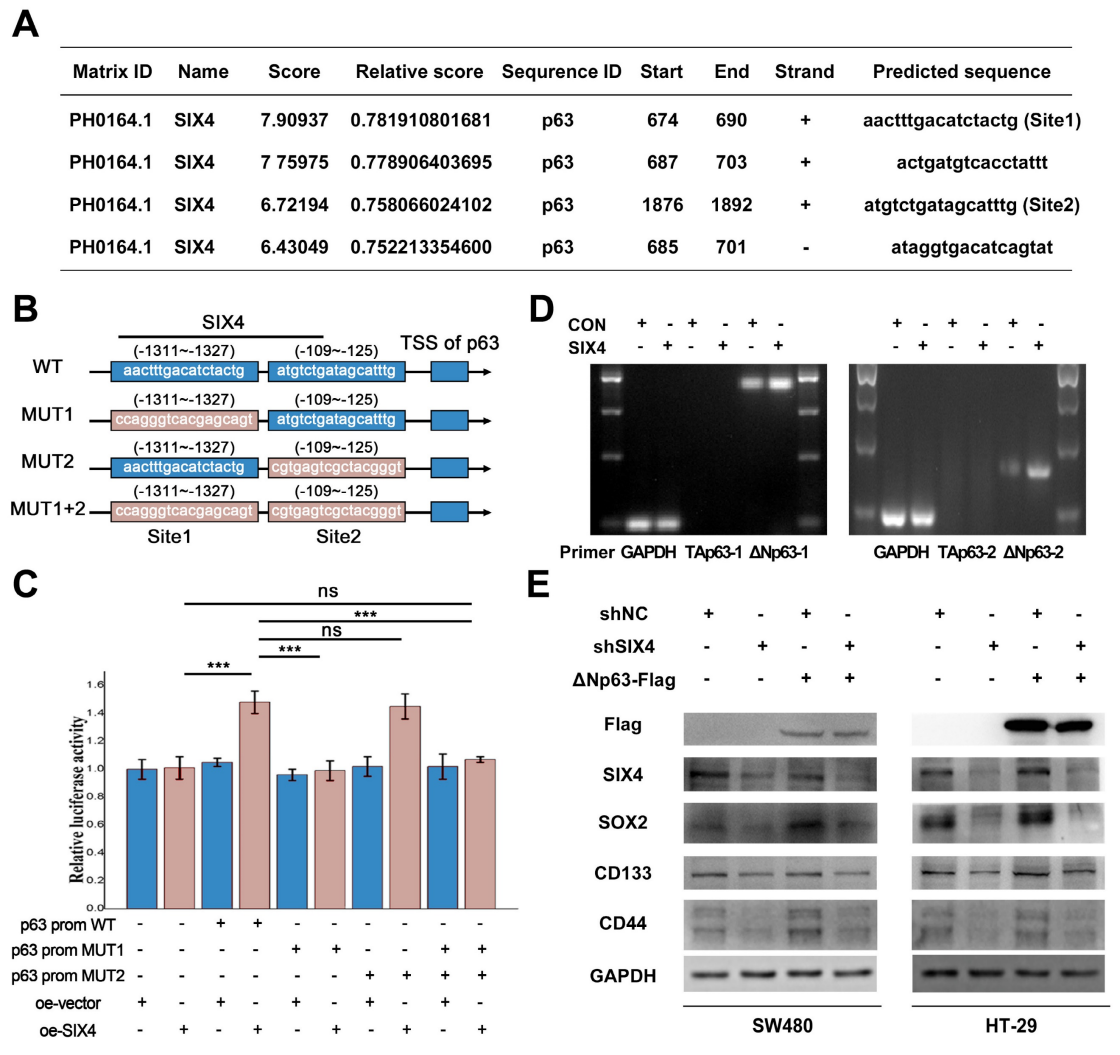
** SIX4 promotes the stemness of colon cancer cells via regulating p63 transcription.** (A) SIX4 binding sites in the promoter of p63 were predicted via JASPAR. (B) Diagram of wild-type and mutant of SIX4 combined with p63 promoter region. (C) The luciferase reporter assay was utilized to assess the role of SIX4 in the transcriptional regulation of p63. (D) Agarose gel electrophoresis analysis were used to detect TAp63 and DeltaNp63 in SIX4 overexpressed cell. (E) Western blot experiments were showed the protein of SIX4, SOX2, CD133, CD44 in NC and SIX4 knockdown cell lines with or without DeltaNp63 plasmids.

Previous studies have reported that SIX4 acts as a transcription factor in liver cancer^35^. In addition, immunohistochemistry (Figure 3B) showed that nuclear expression of SIX4 is increased in tissues with high expression of SIX4. Immunofluorescence experiments (Figure 6C) showed that SIX4 is mainly expressed in the nucleus in the colorectal cancer cell line. Therefore, we speculated that SIX4 may contribute to tumor stemness through transcriptional regulation. In this study, we utilized the JASPAR database to conduct a search for stemness markers that may be modulated by SIX4. As a result, p63 was screened out (Figure 7A-B). Further we designed a dual luciferase expression reporter system, utilizing both wild-type and mutant constructs, which was designed based on the predicted sites (Figure 7B, MUT). The results indicate that SIX4 facilitated the transcription of p63 (Figure 7C). In order to clear the role of SIX4 in relation to Tap63 and DeltaNp63, we conducted an analysis of the expression of these two isotypes in cells overexpressing SIX4. Results showed that the over-expression of SIX4 mainly promoted the expression of DeltaNp63 whereas TAp63 presented extremely low or no expression (Figure 7D). Additionally, the experimental results showed that the overexpression of TAp63 significantly inhibited the expression of tumor stem-related genes OCT4, Nanog, and CD133 (Supplementary Figure 7). Finally, we overexpressed DeltaNp63 in knockdown SIX4 cell lines and found that the overexpression of DeltaNp63 reversed the suppression of SOX2, CD133, and CD44 expression induced by SIX4 knockdown (Figure 7E).

## Discussion

CAC is a major complication and leading cause of death associated with IBD. Although certain genes have been verified to play a role in the progression of this particular form of colorectal cancer, they are insufficient to fully elucidating the underlying mechanisms of CAC. Here, we developed a mouse model of IBD induced by DSS, as well as a combination model of CAC induced by AOM and DSS. We then utilized microarray sequencing to identify genes that were progressively upregulated or downregulated in these models. Due to the lack of human CAC sample and **its** public databases, we had to use the total COAD database to screen candidate genes to uncover the molecular mechanisms shared between CRC and CAC. A comparison was made between 30 progressively up-regulated and 15 genes that were found to be progressively down-regulated through sequencing, using the Colon adenocarcinoma (COAD) TCGA database. Among the genes analyzed, SP5, TREX2, SIX4, and SPP1 exhibited up-regulation in COAD according to the TCGA database. Furthermore, these genes were confirmed to be progressively up-regulated in samples from Control, DSS-induced colitis, and DSS plus AOM model. Consistent with the sequencing results, the overexpression of SP5 has been reported in various human cancers, including hepatocellular carcinoma, gastric cancer, and colon cancer^44^, ^45^. Elevated levels of SPP1 have been found to promote colitis and stimulate tumor growth^46^; Additionally, SIX4 has been reported to be upregulated in osteosarcoma, hepatocellular carcinoma, non-small-cell lung cancer, and colorectal cancer, and it has been shown to promote tumor progression^36^, ^47^, ^48^. Among them, the expression difference of SIX4 was most obvious between normal colorectal tissue and COAD tissue in TCGA database.

Given that CAC is a specific subtype of CRC in humans, despite their distinct etiology, clinical features, and molecular characteristics, the scarcity of clinical specimens for CAC drives our primary focus towards exploring the potential common role of SIX4 in CRC specimens. We have detected the expression of SIX4 in normal intestinal mucosa, IBD tissue, para-cancer tissue, and CRC tissue. Results indicate a significant upregulation of SIX4 in IBD and CRC tissues, when compared to normal mucosa and para-cancer tissues, respectively. Further analysis of bioinformatic and clinical data revealed a significant correlation between the expression of SIX4 and tumor recurrence. Moreover, it was observed that high levels of SIX4 expression were indicative of a poor prognosis in patients. However, the function of SIX4 in inflammation and tumor remains to be further investigated.

To clarify the function of SIX4 in inflammation, we treated normal intestinal epithelial cell lines with LPS and found that gradient concentrations of LPS induced an increase in SIX4 expression. Further, we used *in vivo* siRNA therapy (polyethyleneimine based transport systems) in mice models of DSS-induced colitis and AOM plus DSS-induced colon cancer to clarify the function of SIX4 in the progression of IBD and CAC. Our study findings suggest that inhibition of SIX4 expression can alleviate the changes in colon length induced by DSS, reduce the number of polyps induced by AOM+DSS, and also alleviate the DAI score in both models. Overall, inhibition of SIX4 expression in IBD and CAC models demonstrated a reduction in inflammation and the transformation of inflammation into cancer.

Although we demonstrated that SIX4 is involved in inflammation tumor transformation, the underlying mechanism remains unclear. The animal model effectively replicates the progression from normal tissue to inflammation to tumor and the animal IBD/CAC model has some similarities with human IBD/CAC pathological changes, but studies have shown that the gene changes of IBD in animal model and human are still quite different^49^. To enhance the relevance of the sequencing data to human disease, we conducted an integration of animal model sequencing data with human IBD clinical sequencing data. Through this analysis, we discovered that SIX4 potentially exerts its function by activating the IL6/STAT3 signaling pathway. Cytokines have a significant impact on the pathogenesis of IBD and colorectal cancer. Consistent with previous studies, the levels of IL-6 were found to be significantly elevated in our IBD/CAC model^50^. The JAK/STAT3 signaling pathway exhibits extensive overactivation in both inflammation and tumor. This pathway is regulated by several factors, including IL-6. In this study, we found that the knockdown SIX4 significantly inhibited the expression of IL-6 and reduced the activation of the JAK/STAT3 signaling pathway. BioGrid analysis suggests a potential interaction between SIX4 and C-JUN. It is worth mentioning that C-JUN, as a subunit of AP-1, is considered to promote IL-6 expression. The experiments showed that C-JUN and SIX4 colocalized in the nucleus and exhibited an interaction. Overexpression of the transcription factor SIX4 facilitated the interaction between C-JUN and the promoter region of IL-6. The upregulation of IL-6 induced by the introduction of SIX4 was reversed by interfering with C-JUN. Intriguingly, recombinant human IL-6 stimulated SIX4 expression in colorectal cancer cell lines. Analysis of ChIP-Seq data based on breast cells suggests that STAT3 may regulate SIX4 transcription. Finally, the experimental findings provide confirmation that SIX4 plays a role in promoting the transcription of IL-6 by recruiting C-JUN. Furthermore, the upregulation of IL-6 leads to the activation of JAK/STAT3, which in turn promotes the expression of SIX4. This process establishes a self-activating positive feedback loop and maintains the inflammatory state. However, the reasons for the transition from a state of persistent inflammation to tumorigenesis remain to be further studied.

There is a group of tumor cells with characteristics of stem cells known as CSCs. CSCs were first identified in leukemia, and subsequent studies have shown their presence in a variety of solid tumors, including colorectal cancer^51^, ^52^. The relationship between the immunohistochemical staining score of SIX4 in tissue microarray and clinical parameters suggests that SIX4 expression is associated with colorectal cancer recurrence, and tumor stem cells have been extensively studied as the key factor of tumor recurrence. Therefore, we speculated that SIX4 may be involved in the inflammation and tumor transformation by enhancing the stemness of tumor cells. In subsequent experiments, we found that SIX4 was significantly upregulated in tumorspheres compared to adherent cells. Furthermore, the knockdown of SIX4 resulted in the inhibition of tumor stemness both *in vivo* and *in vitro*. The SIX protein family comprises a set of evolutionarily conserved transcription factors. SIX4 has been reported to function as a transcription factor regulator in liver cancer^35^. Therefore, it is plausible that SIX4 may be involved in the regulation of tumor stemness as a transcription factor. However, what genes does SIX4 regulate to induce tumor stemness in colorectal cancer?

63 is a member of the p53 family. Full-length p63 (TAp63) has a similar domain to p53 and has been reported to induce aging and inhibit tumor initiation and metastasis^53^, ^54^. Compared with TAp63, DeltaNp63 lacks the N-terminal trans-activating domain (TAD). Some studies have shown that DeltaNp63, as a potential oncogene, has been reported to promote the progression of various tumors, including breast cancer, lung cancer, and colorectal cancer, via inducing cell cycle, EMT, and tumor stemness signals^55^-^57^. In addition, DeltaNp63 is important for colorectal epithelial stem/progenitor cells^58^. These data suggest that TAp63 and DeltaNp63 play vastly different roles in tumors.

Interestingly, TAp63 and DeltaNp63 share a common promoter to modulate their respective expressions. Here, we predicted SIX4 as the transcription factor of p63 based on JASPAR database analysis. Subsequently, we observed that SIX4 can bind to the promoter of p63 and induce DeltaNp63 expression, but not TAp63 in colorectal cancer. Moreover, TAp63 expression was hardly detected in colorectal cancer cell lines, which is consistent with the findings in breast cancer^59^. Finally, we found that the overexpression of DeltaNp63 reversed the suppression of SOX2, CD133, and CD44 expression induced by SIX4 Suppression. These findings elucidate a mechanism whereby SIX4 promotes DeltaNp63 transcription by binding to the p63 promoter region, thereby increasing tumor stemness.

In summary, this study demonstrates a progressive upregulation of SIX4 expression, commencing in normal tissue, progressing through inflammatory tissue, and culminating in tumor tissue. Targeting SIX4 alleviates both inflammatory bowel disease (IBD) and colorectal cancer (CAC) development *in vivo*. Furthermore, we confirmed that knocking down SIX4 can inhibit the tumor stem cell phenotype in colorectal cancer (CRC). The underlying mechanism reveals that inflammation facilitates the activation of SIX4 via the IL-6/STAT3 signaling pathway. Additionally, SIX4 promotes its own expression by recruiting C-JUN to activate the IL-6/STAT3 signal, thereby establishing a positive feedback loop. Moreover, upregulated SIX4 enhances the stemness of colon cancer cells by promoting the DeltaNp63 signal, which further accelerates tumorigenesis in CRC.

## Conclusions

This study demonstrates the important role of SIX4 in inflammatory cancer transformation for the first time and intends to provide new prevention and treatment strategies for IBD and CRC patients.

## Supplementary Material

Supplementary figures and table.

## Figures and Tables

**Table 1:** Correlation between the clinicopathologic characteristics and SIX4 expression level in colorectal cancer

Characteristic		Low expression	High expression	Chi-square value	*P* value
Age	<50	10	8	1.316	0.251
	≥50	40	37		
Gender	Female	27	23	0.079	0.778
	Male	23	22		
Recurrence	No	41	28	4.661	**0.031**
	Yes	9	17		
T stage	T1-T2	5	5	0.031	0.86
	T3-T4	45	40		
N stage	N0-N1	31	29	0.061	0.805
	N2-N3	19	16		
M stage	M0	32	33	0.955	0.328
	M1	18	12		
Clinical stage	I-II	23	22	0.079	0.778
	III-IV	27	23		
Pathology grade	I-II	41	41	1.103	0.294
	III-IV	9	4		
Lymphatic metastasis	No	30	30	0.452	0.501
	Yes	20	15		

## Abbreviations

AOM: azoxymethane
CAC: colitis-associated colorectal cancer
CCSCs: colon cancer stem cells
CD: Crohn's disease
ChIP: chromatin immunoprecipitation
COAD: colon adenocarcinoma
Co-IP: co-immunoprecipitation assay
CRC: colorectal cancer
CSCs: cancer stem cells
DAI: disease activity index
DSS: dextran sodium sulfate
FBS: Fetal bovine serum
GFP: green fluorescent protein
HE: hematoxylin and eosin
IBD: inflammatory bowel disease
IF: immunofluorescence staining
LPS: lipopolysaccharide
NC: negative control
PBS: phosphate buffer saline
RT: room temperature
RT-qPCR: reverse transcription-real-time quantitative PCR
UC: ulcerative colitis
NC: Negative control
