# Supplementary material for: SIX4 Activation in Inflammatory Response Drives the Transformation of Colorectal Epithelium into Inflammation and Tumor via Feedback-Enhancing Inflammatory Signaling to Induce Tumor Stemness Signaling
Source: Int J Biol Sci. 2024 Aug 26;20(12):4618–34. doi: 10.7150/ijbs.93411 (PMC11414381; doi:10.7150/ijbs.93411)
Supplement: Supplementary file 1 — Supplementary figures and table. [file ijbsv20p4618s1.pdf]

Title: Activation In Inflammatory Response Drives The Transformation Of Colorectal Epithelium Into Inflammation And Tumor Via Feedback-Enhancing Inflammatory Signaling To Induce Tumor Stemness Signaling

Supplementary Table 1

The primers used in this study

Name	Sequence
SP5	Forward: 5′- TCTAGGCGGGTACTTTGAGC -3′
	Reverse: 5′- TAGGTCTGACTCGCACCCTT -3′
SIX4	Forward: 5′- TTGCTGGATTCATCCTCGGT -3′
	Reverse: 5′- ACAAATGTCTTCTCCTCCCCCT -3′
TREX2	Forward: 5′- AGGCCCTGCTACCTATAGCC -3′
	Reverse: 5′- GAGATGGGATGGAGCATCAGG -3′
SPP1	Forward: 5′- CGTCCCTACAGTCGATGTCC -3′
	Reverse: 5′- TCAAGCCATAGCCCTTCAACA -3′
B3GNT7	Forward:5′- CCATGTCTCTGTGGTGAGTCG -3′
	Reverse:5′- CCATGTCTCTGTGGTGAGTCG -3′
GAL3ST2	Forward:5′- CCCACCTGCAGCATCACTTT -3′
	Reverse:5′- GCCCCATAGTTACACCCACA -3′
GAPDH	Forward: 5′- CTGGAACTCACCCGTTCACA -3′
	Reverse: 5′- CTGGAACTCACCCGTTCACA -3′

Supplementary Figure 1

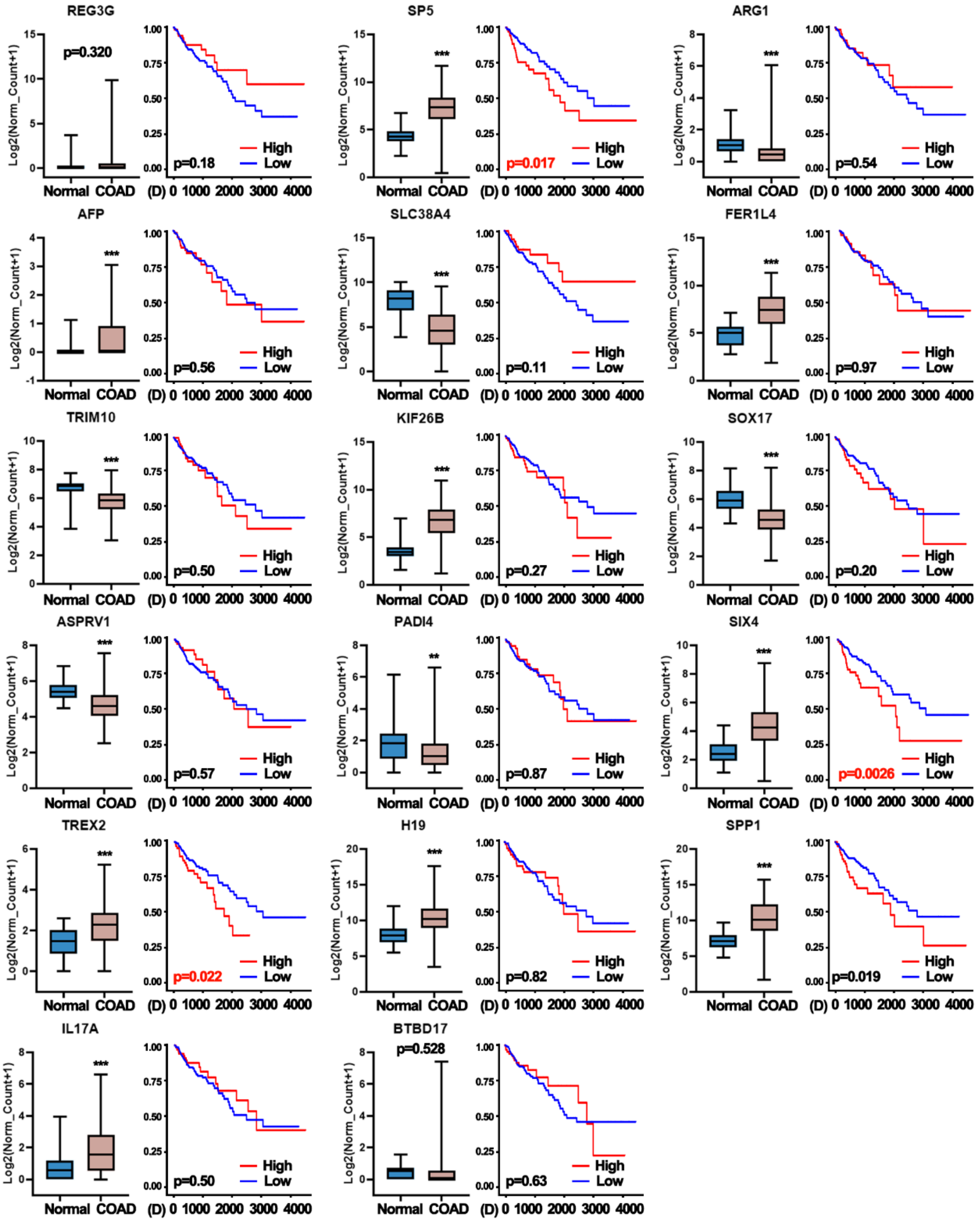

Fig. S1 Gradient up-regulated gene expression in normal intestinal tissues, IBD tissues and CAC tissues and its relationship with overall survival of patients with CRC. Gene expression data came from TCGA database and survival analysis data came from UALCAN database.

Supplementary Figure 2

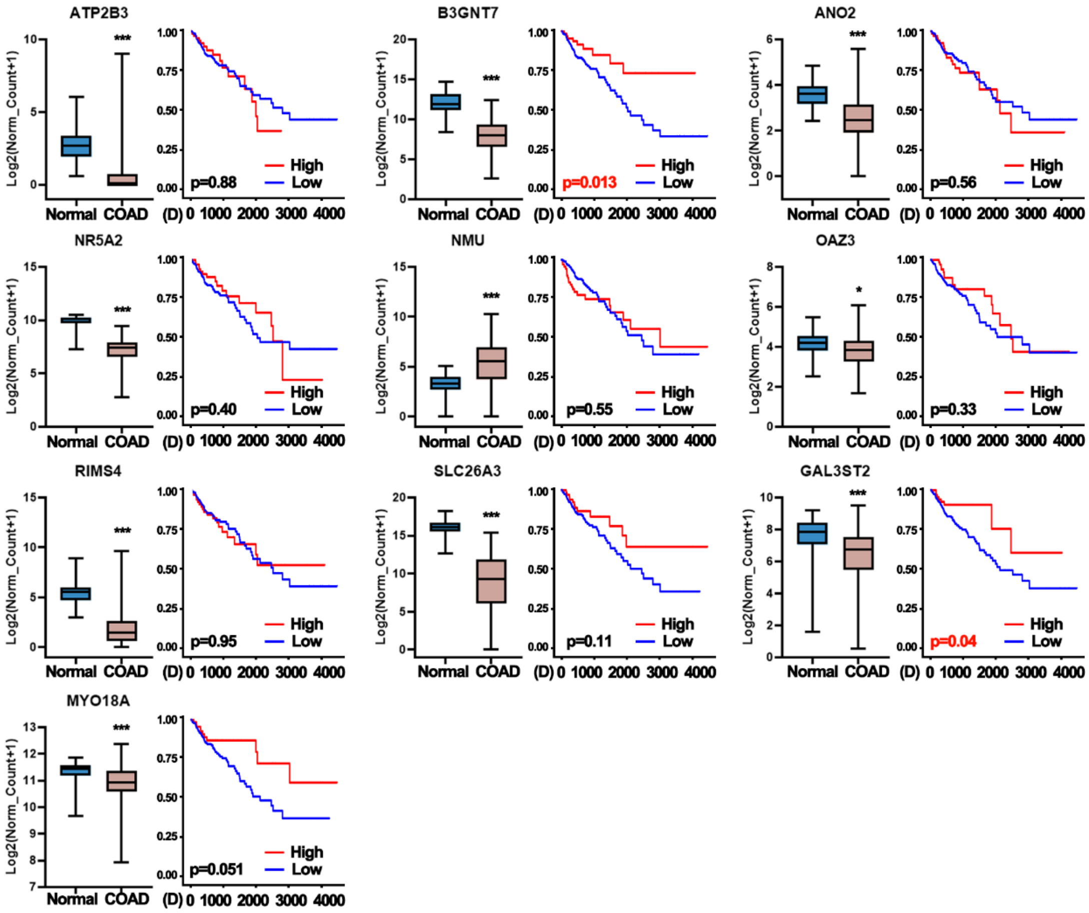

Fig. S2 Gradient down-regulated gene expression in normal intestinal tissues, IBD tissues and CAC tissues and its relationship with overall survival of patients with CRC. Gene expression data came from TCGA database and survival analysis data came from UALCAN database.

Supplementary Figure 3

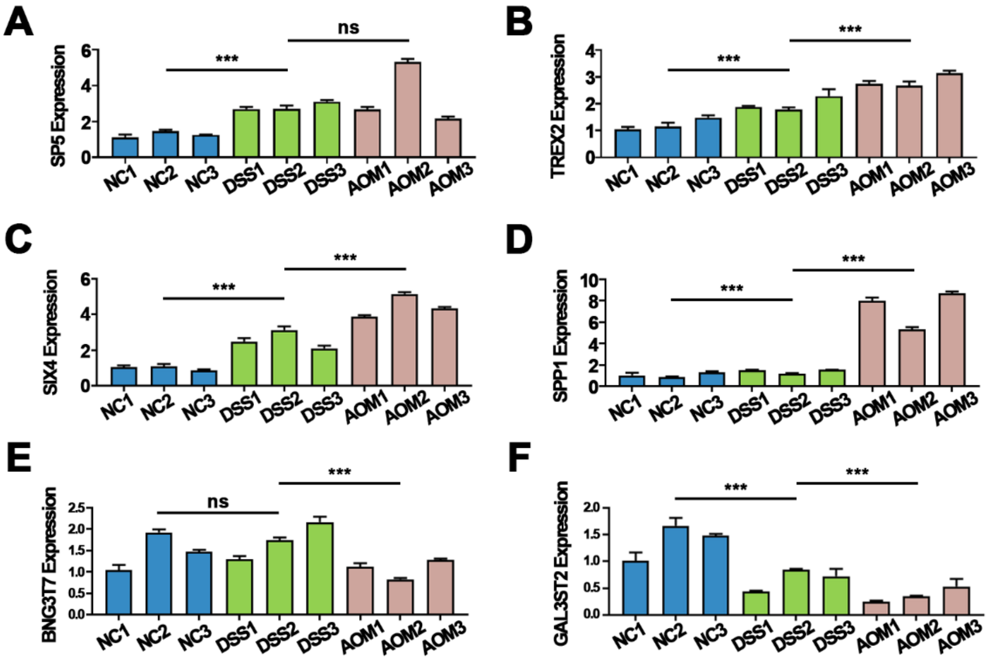

Fig. S3 Expression of candidate genes in control group, DSS induced IBD model group and AOM+DSS induced CAC tissues. The colorectal tissues of control group mice, DSS treated mice and AOM+DSS treated mice were cleaved respectively to extract total RNA and reverse transcribed into cDNA. qPCR was used to verify whether the candidate genes had gradient changes in normal tissues, IBD tissues and CAC tissues.

Supplementary Figure 4

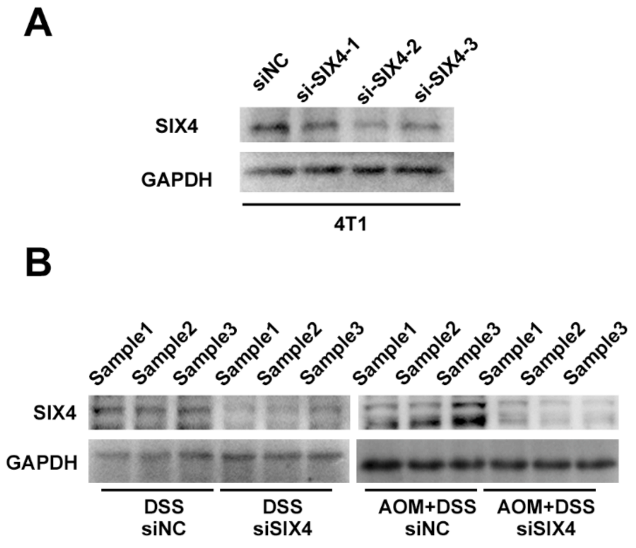

Fig. S4 Screening and efficiency verification of siRNA sequences in animals. (A) Three murine SIX4-specific siRNA sequences were constructed and transfected into murine breast cancer cell line 4T1. The knockdown efficiency was detected after protein extraction, and the si-SIX4-2 sequence had the highest knockdown efficiency. si-SIX4-2 was therefore used in subsequent animal experiments. (B) After the mice were treated in vivo siNC/siSIX4 according to Fig2C/2G, mouse colorectal tissue was harvested and proteins were extracted to verify the SIX4 knockdown efficiency

Supplementary Figure 5

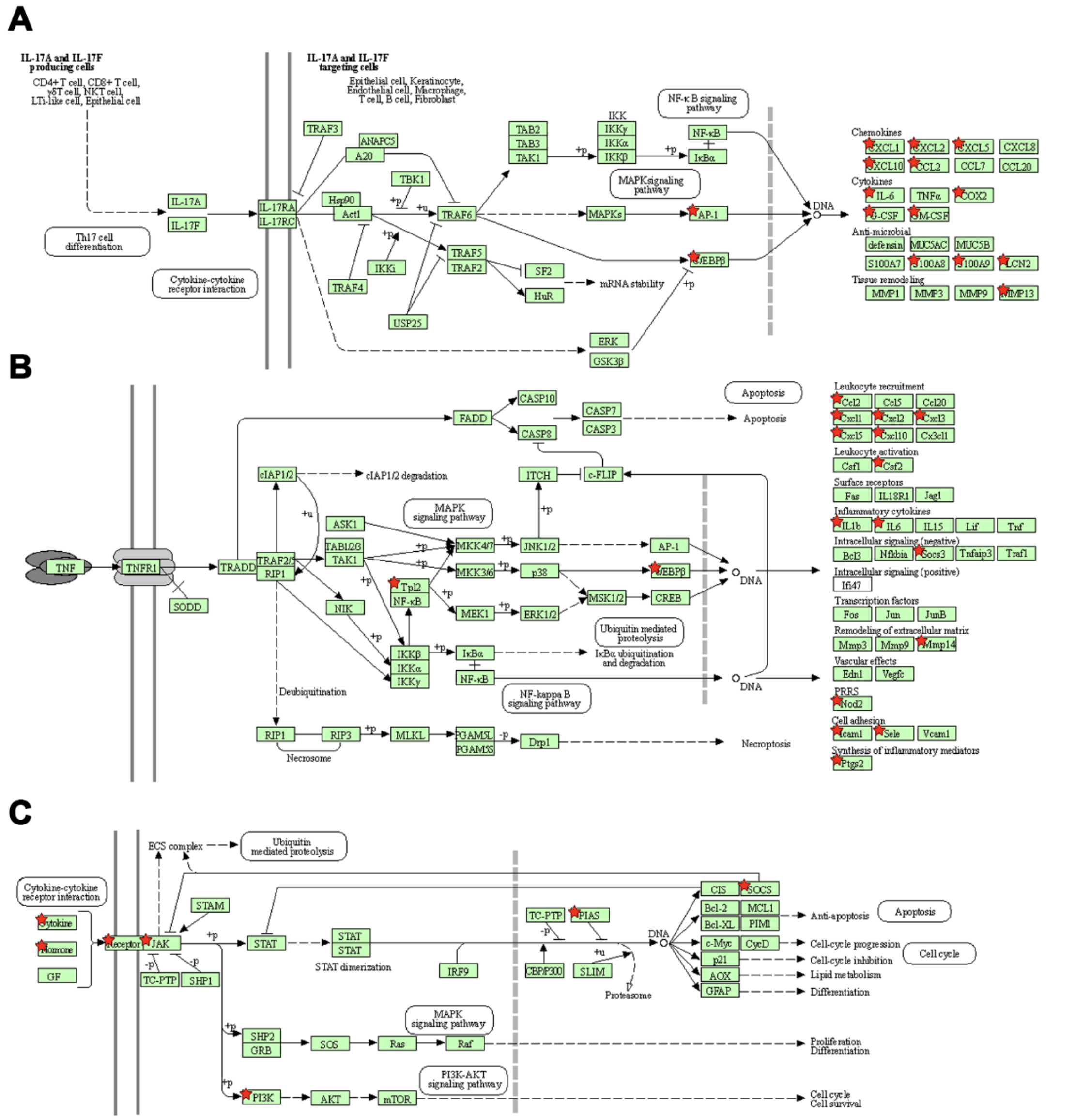

Fig. S5 KEGG pathway enrichment tool was used to analyze the distribution of intersection genes in signaling pathways. (A) IL-17 signaling pathway and (B) TNF signaling pathway eventually promote the expression of cytokines and their receptors, which converge to (C) Cytokine-cytokine receptor interaction pathway and thus activate the JAK/STAT signaling pathway, which in turn leads to cascade activation of the downstream MAPK and PI3K/AKT signaling pathways.

Supplementary Figure 6

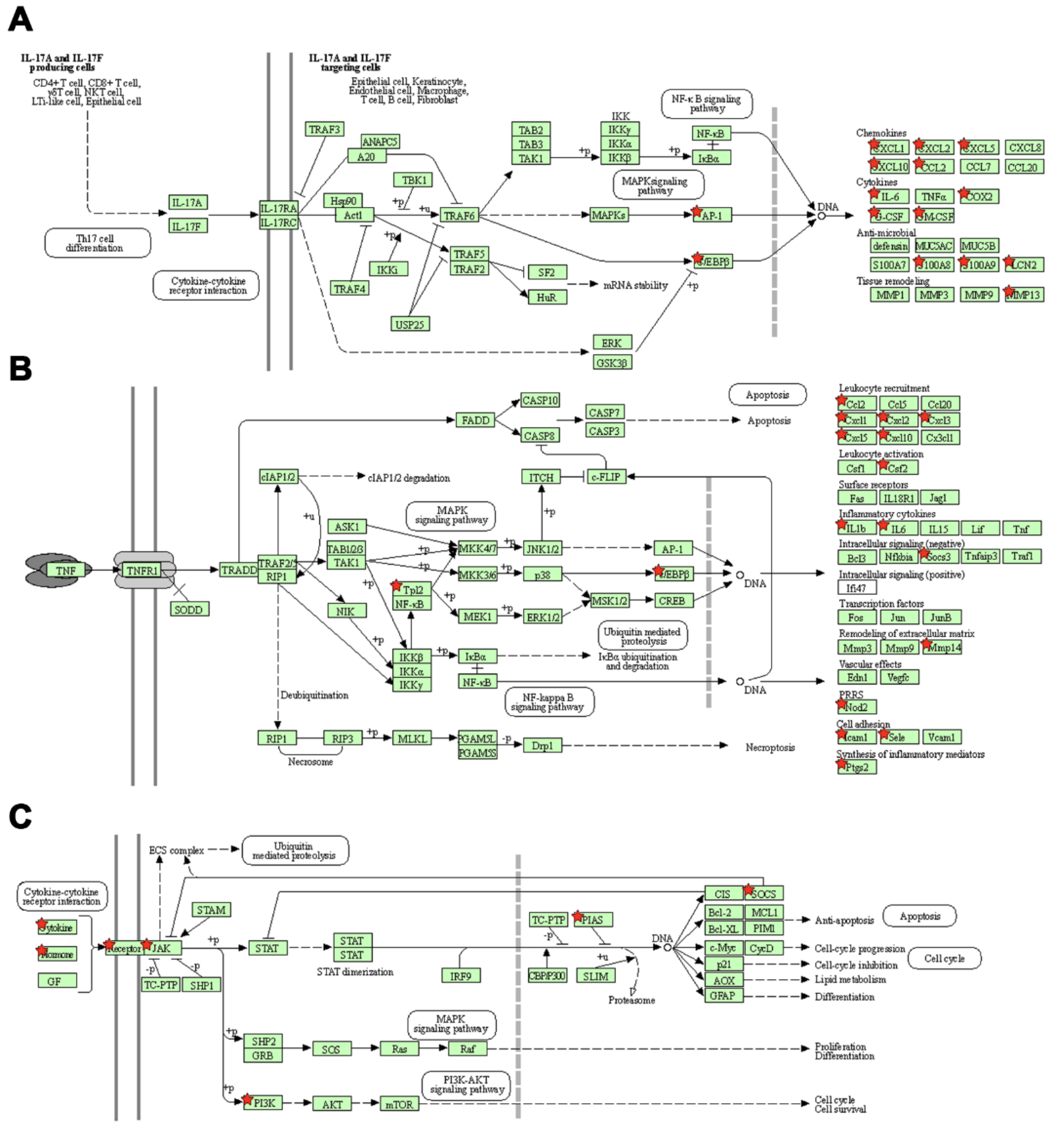

Fig. S6 The database predicts that STAT3 is bound to the SIX4 promoter region. (A) The ChIP-Seq dataset collected by ENCODE database showed that STAT3 had a binding peak in the SIX4 promoter region in MCF10A cell line (Data set numbers are indicated in the graph). (B) The JASPAR database predicts the possible binding sites of STAT3 within 2000bp upstream of the SIX4 coding region.

Supplementary Figure 7

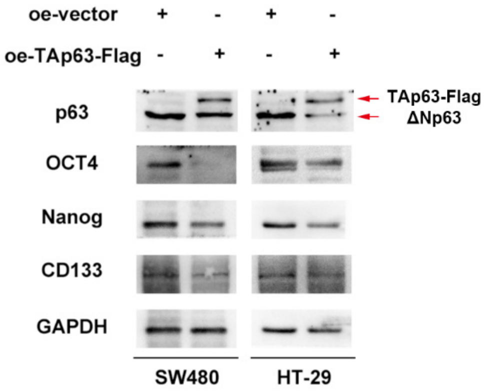

Fig. S7 Overexpression of TAp63 inhibited tumor stemness. The overexpression of TAp63-Flag in SW480 and HT-29 cell lines significantly inhibited the expression of tumor stemness markers OCT4, Nanog and CD133.
